# Mitigation of Glucolipotoxicity-Induced Apoptosis, Mitochondrial Dysfunction, and Metabolic Stress by *N*-Acetyl Cysteine in Pancreatic β-Cells

**DOI:** 10.3390/biom10020239

**Published:** 2020-02-05

**Authors:** Arwa Alnahdi, Annie John, Haider Raza

**Affiliations:** Department of Biochemistry, College of Medicine and Health Sciences, United Arab Emirates University, P.O. Box 17666, Al Ain, UAE; 200770005@uaeu.ac.ae (A.A.); anniej@uaeu.ac.ae (A.J.)

**Keywords:** glucolipotoxicity, palmitic acid, Rin-5F cells, mitochondrial dysfunction, autophagy, apoptosis

## Abstract

Glucolipotoxicity caused by hyperglycemia and hyperlipidemia are the common features of diabetes-induced complications. Metabolic adaptation, particularly in energy metabolism; mitochondrial dysfunction; and increased inflammatory and oxidative stress responses are considered to be the main characteristics of diabetes and metabolic syndrome. However, due to various fluctuating endogenous and exogenous stimuli, the precise role of these factors under in vivo conditions is not clearly understood. In the present study, we used pancreatic β-cells, Rin-5F, to elucidate the molecular and metabolic changes in glucolipotoxicity. Cells treated with high glucose (25 mM) and high palmitic acid (up to 0.3 mM) for 24 h exhibited increased caspase/poly-ADP ribose polymerase (PARP)-dependent apoptosis followed by DNA fragmentation, alterations in mitochondrial membrane permeability, and bioenergetics, accompanied by alterations in glycolytic and mitochondrial energy metabolism. Our results also demonstrated alterations in the expression of mammalian target of rapamycin (mTOR)/5′ adenosine monophosphate-activated protein kinase (AMPK)-dependent apoptotic and autophagy markers. Furthermore, pre-treatment of cells with 10 mM *N*-acetyl cysteine attenuated the deleterious effects of high glucose and high palmitic acid with improved cellular functions and survival. These results suggest that the presence of high energy metabolites enhance mitochondrial dysfunction and apoptosis by suppressing autophagy and adapting energy metabolism, mediated, at least in part, via enhanced oxidative DNA damage and mTOR/AMPK-dependent cell signaling.

## 1. Introduction

Prolonged hyperglycemia and hyperlipidemia induces metabolic adaptation, particularly in energy metabolism, leading to alterations in insulin secretion, signaling, and insulin resistance, as seen in diabetes and obesity (diabesity)-associated complications [1,2,3,4]. Metabolic stress, inflammatory responses, and oxidative stress have been implicated in the etiology and pathology of cancer, diabetes, cardiovascular disorders, and numerous other metabolic disorders. Studies have suggested that dietary management is helpful in reducing the complications of these diseases [5,6]. Nutrient overload, such as excess of glucose and fatty acids, under in vivo (as seen in obesity and diabetes) and in vitro conditions, which induces inflammatory and oxidative stress responses, may be the key factors in inducing mitochondrial dysfunction, resulting in disease development and progression [7,8,9,10,11]. Once the primary pathogenesis of diabetes is established, which is potentially linked to both genetic and environmental factors, hyperglycemia and hyperlipidemia exert further deleterious effects on metabolic responses in different tissues. Owing to the differential uptake, storage, and hormonal regulation of glucose and fatty acid metabolism in different tissues, and to avoid the interference of endogenous (autocrine/endocrine/growth factors/cytokines) and exogenous (mainly dietary) responses in vivo, we designed a cell model to investigate the molecular mechanism of gluco/lipotoxicity. Our recent study on insulin sensitive/target HepG2 cells demonstrated that high glucose (25 mM) and high palmitic acid (up to 0.3 mM) exhibit marked induction in oxidative stress, and alterations in inflammatory and metabolic signaling causing mitochondrial dysfunction and cytotoxicity [12].

Because inflammation, oxidative stress, and mitochondrial dysfunction appear to be involved in inducing apoptosis and thus cytotoxicity in tissues, there might be common molecular mechanisms involved in the etiology and pathophysiology of glucolipotoxicity. We, therefore, extended our study to insulin-secreting pancreatic β-cell, Rin-5F, to investigate the broader mechanism(s) of glucolipotoxicity.

The main objective was to identify the specific molecular and metabolic targets affected under these conditions and to elucidate the protective mechanism(s) of glucolipotoxicity through therapeutic interventions, resulting in better understanding and management of hyperglycemic and hyperlipidemic conditions, observed in diabetes and obesity. In our previous study, we demonstrated increased oxidative and inflammatory stress in Rin-5F cells treated with high glucose (25 mM) and/or high palmitic acid (up to 0.3 mM) [13]. These effects were accompanied by altered glutathione (GSH)-dependent redox homeostasis and antioxidant responses induced by NF-kB. In the present study, we further investigated the mechanism of mitochondrial dysfunction and apoptosis induced in Rin-5F cells treated with high glucose and palmitic acid. Our results demonstrated reduced mitochondrial membrane potential and respiratory bioenergetics in these cells, causing DNA fragmentation and apoptosis. We also observed altered energy metabolism and mammalian target of rapamycin (mTOR)/5′ adenosine monophosphate-activated protein kinase (AMPK)-dependent cell signaling, resulting in inhibition of autophagy. Our study also demonstrated that pre-treatment of cells with *N*-acetyl cysteine (NAC), a GSH precursor and ROS scavenger, attenuated the effects of glucolipotoxicity seen in these cells. 

## 2. Materials and Methods

### 2.1. Materials

Fatty acid-free bovine serum albumin (BSA), palmitic acid, *N*-acetyl cysteine (NAC), L-glucose, NADH, NADPH, cytochrome c, coenzyme Q2, antimycin A, dodecyl maltoside, Hoechst 33342, and kits for ATP and hexokinase (HK) were purchased from Sigma (St. Louis, MO, USA). Kits for caspase-3 and -9 assays and for mitochondrial membrane potential assays were purchased from R&D Systems (Minneapolis, MN, USA) and that for aconitase from Oxis Int, Inc. (Portland, OR, USA). Apoptosis detection kits for flow cytometry were purchased from BD Pharmingen (BD Biosciences, San Jose, CA, USA) and for immunocytochemistry (transferase-mediated deoxyuridine triphosphate nick end labeling [TUNEL]assay) from Chemicon International, Inc. (Temecula, CA, USA). Kits for glutamate dehydrogenase (GDH) were purchased from Abcam (Cambridge, United Kingdom). Rin-5F cells were obtained from the American Type Culture Collection (Manassas, VA, USA). Polyclonal antibodies against poly-ADP ribose polymerase (PARP), cytochrome c (cyt c), and β-actin were purchased from Santa Cruz Biotechnology Inc. (Santa Cruz, CA, USA), whereas those against autophagy-related gene 5 (Atg5), microtubule-associated protein 1A/1B-light chain 3LC3, voltage-dependent anion channel (VDAC), AMPK, phosphorylated 5′ adenosine monophosphate-activated protein kinase (p-AMPK), mTOR, and phosphorylated mammalian target of rapamycin (p-mTOR) were purchased from Cell Signaling Technology, Inc. (Danvers, MA, USA). Reagents for cell culture, SDS-PAGE, and Western blot analyses were purchased from Gibco BRL (Grand Island, NY, USA) and Bio Rad Laboratories (Richmond, CA, USA).

### 2.2. Methods

#### 2.2.1. Cell Culture and Treatment 

Rin-5F cells were grown in poly-l-lysine coated 75 cm^2^ flasks (≈2.0–2.5 × 10^6^ cells/mL) in RPMI-1640 medium supplemented with 10% fetal bovine serum (FBS) and 1% non-essential amino acids in a humidified incubator in the presence of 5% CO_2_ at 37 °C. Cell cultures, on reaching 80% confluence, were treated with normal (11 mM) or high glucose (25 mM) alone or in the presence of palmitic acid (0.06 mM and 0.3 mM) for 24 h (stock solution of palmitic acid (100 mM) was prepared in warm ethanol and then conjugated to fatty acid free-BSA in a molar ratio of 6:1). Control cells for normal and high glucose media were treated with vehicle (BSA/ethanol alone, the maximum ethanol concentration being <0.01%). To confirm that the effects of high glucose concentration were not associated with osmotic stress, which could affect cell morphology and functions, effects of non-metabolizable l-glucose (25 mM) as an osmotic control were also studied (data not shown). Concentrations and time points were based on 3-(4,5-dimethylthiazol-2-yl)-2,5-diphenyltetrazolium bromide (MTT) cytotoxicity tests and previously published reports [12,13,14,15]. For NAC treatment, cells were treated with 10 mM NAC, 2 h prior to palmitic acid treatment. After the desired time of treatment, cells were harvested, washed with phosphate-buffered saline (PBS, pH7.4) and homogenized in H-medium buffer (70 mM sucrose, 220 mM mannitol, 2.5 mM HEPES, 2 mM EDTA (ethylenediaminetetraacetic acid), 0.1 mM phenylmethylsulphonylfluoride, pH 7.4) at 4 °C. Mitochondrial and post-mitochondrial fractions were then isolated by differential centrifugation, as described earlier [12,16,17]. Protein concentration was determined using the Bradford method [18]. 

#### 2.2.2. Measurement of Apoptosis, DNA Damage, and Caspase Activities

Flow cytometry analysis was performed to measure the apoptosis after treatment of Rin-5F cells with palmitic acid in the presence of normal/high glucose as per the vendor’s protocol (BD Biosciences, San Jose, CA, USA), as described previously [12,16,17,19,20,21]. Briefly, control and treated cells were trypsinized, washed with PBS, and re-suspended (1 × 10^6^ cells/mL) in binding buffer (10 mM HEPES, pH 7.4, 140 mM NaCl, 2.5 mM CaCl2). A fraction (100 µL/1 × 10^5^ cells) of the cell suspension was then incubated with 5 µL annexin V conjugated to fluorescein isothiocyanate (FITC) and 5 µL propidium iodide (PI) for 15 min at 25 °C in the dark. Binding buffer (400 µL) was added to the suspension and apoptosis measured immediately using a Becton Dickinson FACSCanto II analyser (BD Biosciences, San Jose, CA, USA). This method was able to distinguish the viable from the cells undergoing apoptosis/necrosis.

Apoptosis measurement for DNA damage was performed using Hoechst 33342, a blue fluorescent DNA dye that stains condensed chromatin in apoptotic cells [22,23]. Briefly, cells were grown on cover slips and treated with high glucose/high palmitic acid in the presence of normal or high glucose. Cells were then fixed with 3.7% formaldehyde and stained with Hoechst 33342 (10 µg/mL) for 20 min at room temperature. The cover slips were then washed, mounted on glass slides, and analyzed by fluorescence microscopy. DNA damage was also measured by DNA fragmentation using the standard 2% agarose gel electrophoresis and staining the separated fragments with ethidium bromide, as described previously [24,25,26]. To confirm DNA damage by apoptosis, immunocytochemical staining of DNA strand breaks was detected by the TUNEL assay by using the Apop Tag^R^ peroxidase in situ apoptosis detection kit, as per the manufacturer’s instructions. Briefly, cells grown on cover slips were fixed with 3.7% formaldehyde, and the 3′-OH terminals generated by DNA fragmentation were labeled with digoxigenin-nucleotide, and were then allowed to bind to an anti-digoxigenin antibody conjugated to peroxidase. The bound conjugate enzymatically generated a localized stain in the presence of a chromogenic substrate. Methyl green was used as a counter stain to stain the normal nuclei. The coverslips were then mounted on glass slides and observed under a light microscope.

Apoptosis was also assessed by measuring the activities of the caspases -9 and -3 in the cell lysates, using the respective kits as per the vendor’s protocol. The assays were based on the release of a chromophore, p-nitro aniline (pNA), conjugated to caspase-specific substrates, LEHD (substrate with amino acid sequence, Leu-Glu-His-Asp) and DEVD (substrate with amino acid sequence, Asp-Glu-Val-Asp) for caspase-9 and -3, respectively. The light emitted when the chromophore is released was measured at 405 nm using a plate reader, as described in the manufacturer’s protocol (R&D Systems, MN, USA).

#### 2.2.3. Measurement of Mitochondrial Functions 

##### Measurement of Mitochondrial Membrane Potential MMP (Δψm) 

Mitochondrial membrane potential (Δψm) was measured by flow cytometry using a fluorescent cationic dye, DePsipher, according to the vendor’s protocol (DePsipher, R&D System Inc.), as described previously [19,20,21]. The dye readily enters healthy mitochondria and fluoresces red in its multimeric form. However, in apoptotic cells, the mitochondrial membrane potential collapses, and the dye cannot enter the mitochondria and remains in the cytoplasm in its green fluorescent monomeric form.

##### Measurement of Mitochondrial Enzymes and Bioenergetics 

Mitochondrial respiratory enzyme complexes were measured by suspension of the cell extracts from control and palmitic acid-treated cells in 20 mM potassium phosphate buffer, pH 7.4, in the presence of the lauryl maltoside (0.2%). The activities of NADH ubiquinone oxidoreductase (complex I), succinate-ubiquinone oxidoreductase/ubiquinol-cytochrome c oxidoreductase (complex II/III), and cytochrome c oxidase (complex IV) were measured using the substrates coenzyme Q2, succinate, and reduced cytochrome c, respectively, using the methods of Birch-Machin and Turnbull [27], as described previously [25,26,28].

The ATP content in control and palmitic acid-treated Rin-5F cells was measured using the ATP Bioluminescent assay kit according to the manufacturer’s recommendations (Sigma, St Louis, MO, USA) and analyzed using the TD-20/20 Luminometer (Turner Designs, Sunnyvale, CA, USA), as described previously [29]. Krebs’ cycle enzymes, aconitase, and glutamate dehydrogenase were measured using the appropriate kits according to the manufacturer’s protocols, as described previously [21].

#### 2.2.4. Measurement of Activities of Energy Metabolizing Enzymes 

Activity of hexokinase, the initial rate-limiting enzyme in the glycolytic pathway, was measured using the HK assay kit (Sigma-Aldrich, MO, USA) as per the manufacturer’s instructions. Activity of glucose-6-phosphate dehydrogenase (G6PDH) was measured by following the increase in absorbance due to the reduction of NADP to NADPH at 340 nm, using glucose-6-phosphate as substrate.

#### 2.2.5. SDS-PAGE and Western Blot Analysis 

Proteins from total cell extracts (30 µg) or mitochondrial extracts (10 µg) were resolved by 12% SDS-PAGE and electrophoretically transferred on to nitrocellulose membranes by Western blotting, as described previously [19,20,21,30]. The transferred proteins were probed with primary antibodies and immunoreactive protein bands were then visualized using the Typhoon FLA 9500 system (GE Healthcare, Uppsala, Sweden). Beta actin and VDAC were used as loading controls for post-mitochondrial and mitochondrial extracts, respectively. Densitometric analysis was performed and expressed as relative ratios normalized against actin/VDAC or other proteins as appropriate.

#### 2.2.6. Statistical Analysis

Values shown are expressed as mean ± SEM (standard error of mean) of three individual experiments. Statistical significance of the data was assessed using SPSS software (version 23) by analysis of variance followed by least significant difference (LSD) post-hoc analysis. *p*-values ≤ 0.05 were considered statistically significant.

## 3. Results

### 3.1. Effects of High Glucose/High Fatty Acids on Apoptosis

**Figure 1:**
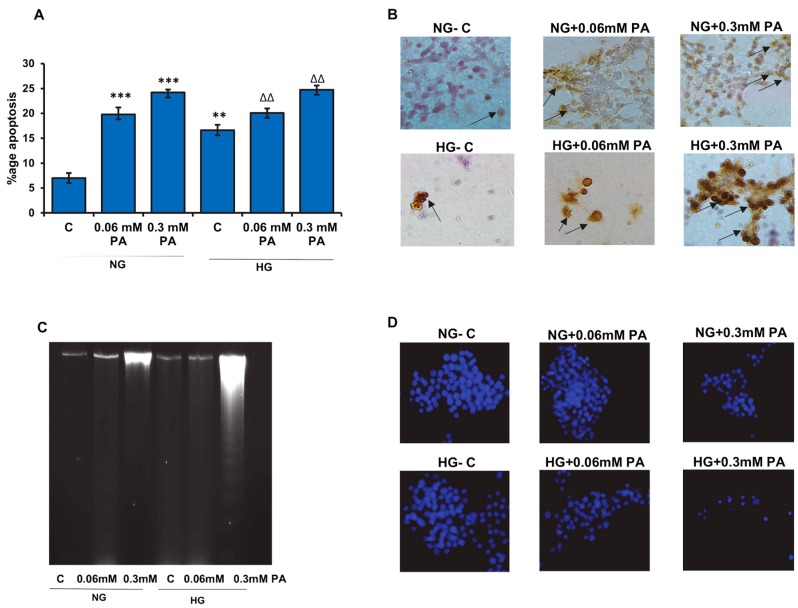
High glucose/high palmitic acid-induced apoptosis in Rin-5F cells. Apoptosis was measured in Rin-5F cells treated with different doses of palmitic acid (PA) under normal (NG) and high glucose (HG) conditions by flow cytometry (**A**). Percentage of apoptotic cells is represented as a histogram. In some cases, cells were grown on coverslips, and immunocytochemical localization of apoptosis (DNA fragmentation) was determined using the Apop Tag peroxidase in situ apoptosis detection kit, as per the manufacturer’s instructions. Some of the apoptotic nuclei are indicated with arrows (**B**). DNA fragmentation was also analyzed by agarose gel (2%) electrophoresis and ethidium bromide staining (**C**). Staining of nuclei of treated cells was also performed using Hoechst33342 dye (**D**) and analyzed by fluorescence microscopy. Cells with signs of apoptosis showed decrease in stained nuclei. Representative slides from three experiments are shown. Original magnification ×200. Asterisks indicate significant differences (** *p* ≤ 0.01, *** *p* ≤ 0.001) relative to untreated control cells under normal glucose condition (NG-C), and triangles indicate significant differences (ΔΔ *p* ≤ 0.01) relative to untreated control cells under high glucose condition (HG-C).

Figure 1A shows the effect of palmitic acid on apoptosis in Rin-5F cells in the presence of normal or high glucose. A significant dose-dependent increase in the percentage (20–25%) of cells undergoing apoptosis was observed. Our results demonstrated that the effect of palmitic acid on apoptosis was augmented in the presence of normal and high glucose.

Palmitic acid treatment also caused an increase in DNA fragmentation under normal as well as high glucose conditions, though it was more pronounced under high glucose condition, as seen by immunocytochemistry as well as by agarose gel electrophoresis (Figure 1B,C). Consistently, a decrease in Hoechst stained nuclei was observed with higher concentration of palmitic acid in the presence of normal and high glucose (Figure 1D).

**Figure 2:**
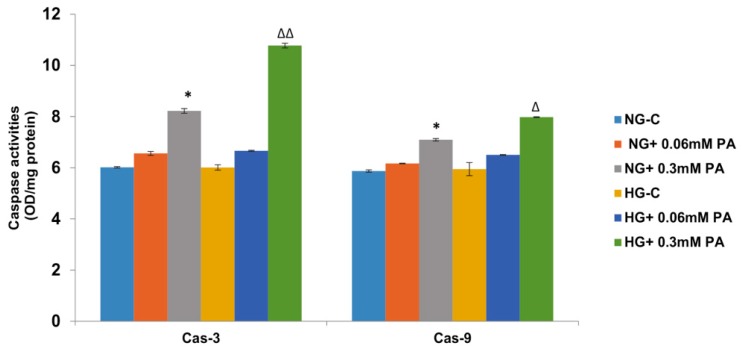
High glucose/high palmitic acid increased the activities of caspase-3 (Cas-3) and -9 (Cas-9). Activities of caspases were measured in treated cells colorimetrically using the respective substrates as described in the Materials and Methods section. Results are expressed as mean +/− SEM of three experiments. Asterisks indicate significant differences (* *p* ≤ 0.05) relative to untreated control cells under normal glucose condition (NG-C), and triangles indicate significant differences (Δ *p* ≤ 0.05, ΔΔ *p* ≤ 0.01) relative to untreated control cells under high glucose condition (HG-C).

Palmitic acid-induced increase in apoptosis was further confirmed by the increase in the activities of caspase-3 and -9 enzymes (Figure 2). A moderate increase in caspase-3 activity was observed with 0.06 mM palmitic acid, which further increased with 0.3 mM palmitic acid (almost 40%) in the presence of normal glucose, which was aggravated in the presence of high glucose. A similar increase was observed with caspase-9 activity.

### 3.2. Effects of High Glucose/High Fatty Acids on Mitochondrial Functions

#### 3.2.1. Effects of High Glucose/High Fatty Acids on Mitochondrial Membrane Potential

**Figure 3:**
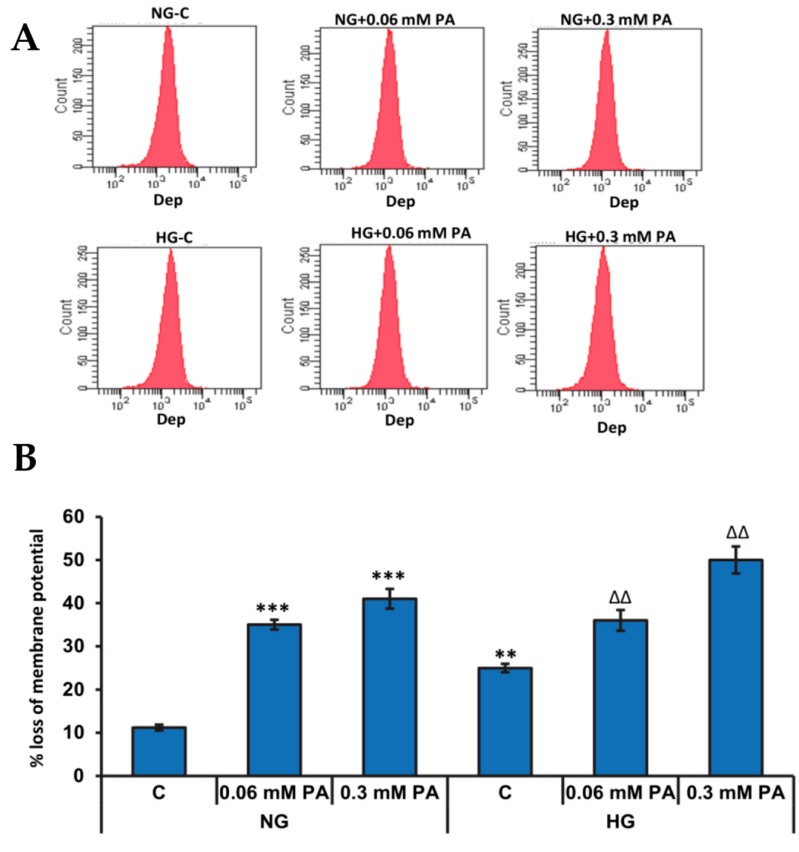
High glucose/high palmitic acid treatment induced alteration in the mitochondrial membrane potential. Mitochondrial membrane potential (Δψm) was measured by flow cytometry (**A**) using a fluorescent cationic dye according to the vendor’s protocol. A typical histogram (**B**) representing the percentage loss of mitochondrial membrane potential is shown. Results are expressed as mean +/− SEM of three experiments. Asterisks indicate significant differences (** *p* ≤ 0.01, *** *p* ≤ 0.001) relative to untreated control cells under normal glucose condition (NG-C), and triangles indicate significant differences (ΔΔ *p* ≤ 0.01) relative to untreated control cells under high glucose condition (HG-C).

The mitochondrial membrane potential (MMP) plays a crucial role in determining the mitochondrial bioenergetics and fate of the cells under conditions of oxidative stress and availability of excess nutrients. Significant loss in the membrane potential was observed after treatment with palmitic acid in the presence of both normal and high glucose in a concentration-dependent manner (Figure 3).

#### 3.2.2. Effects of High Glucose/High Fatty Acids on Mitochondrial Enzymes and Bioenergetics

**Figure 4:**
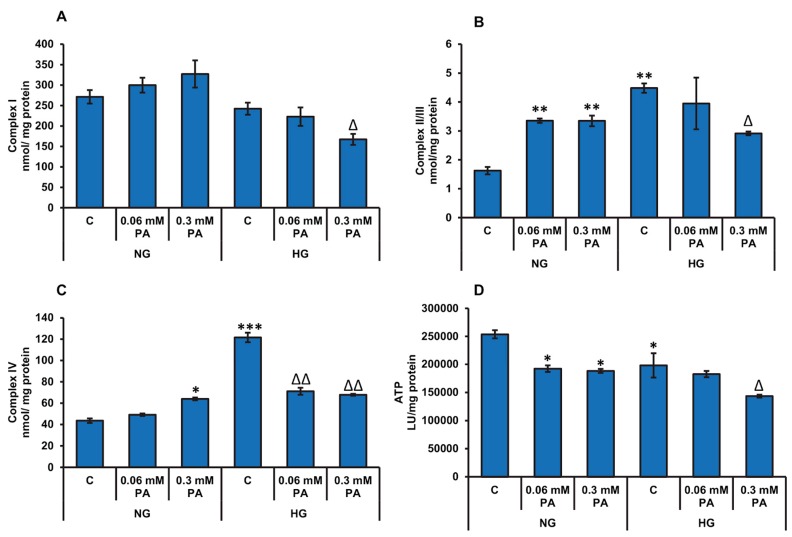
High glucose/high palmitic acid treatment-induced alterations in mitochondrial enzyme activities and ATP production. Rin-5F cells were treated with (0.06 mM and 0.3 mM) palmitic acid under normal and high glucose conditions. Respiratory complex I (**A**), complex II/III (**B**), complex IV (**C**), and ATP (**D**) were measured as described previously in the Materials and Methods section. Results are expressed as mean +/− SEM of three experiments. Asterisks indicate significant differences (* *p* ≤ 0.05, ** *p* ≤ 0.01, *** *p* ≤ 0.001) relative to untreated control cells under normal glucose condition (NG-C), and triangles indicate significant differences (Δ *p* ≤ 0.05, ΔΔ *p* ≤ 0.01) relative to untreated control cells under high glucose condition (HG-C).

Figure 4 shows the effects of high glucose/high palmitic acid treatment on the activities of mitochondrial respiratory enzyme complexes and the ATP production. The palmitic acid treatment caused a mild-to-significant increase in the activities of complexes I, II/III, and IV (Figure 4A–C, respectively) under normal glucose conditions. However, in the presence of high glucose, palmitic acid treatment suppressed the activities of the mitochondrial respiratory complexes. Significant reduction of the mitochondrial complex activities were observed with 0.3 mM palmitic acid at high glucose concentration. A significant inhibition (≈24–40%) in ATP production was also observed under normal glucose conditions after palmitic acid treatment (Figure 4D). However, under high glucose condition, significant inhibition in ATP was observed only with 0.3 mM palmitic acid. High glucose alone also caused a decrease in ATP production, suggesting an adaptation in energy metabolism against the excessive availability of energy nutrients.

**Figure 5:**
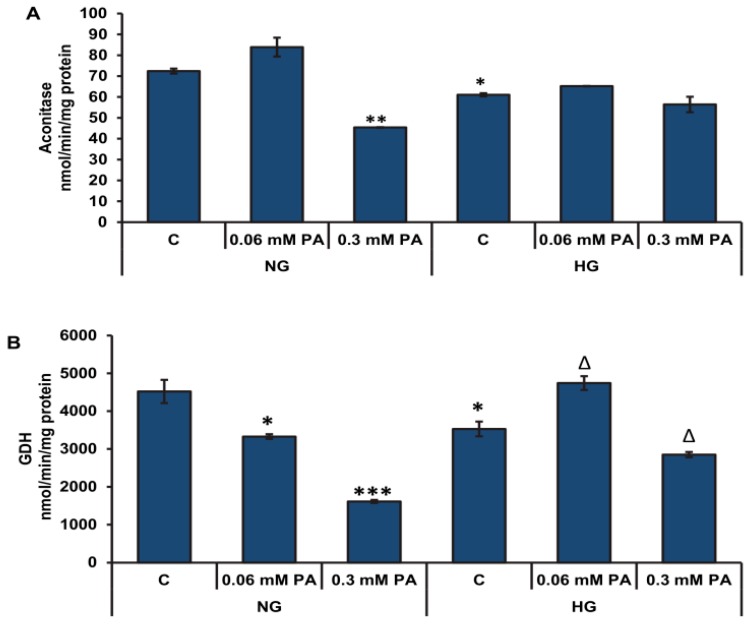
High glucose/high palmitic acid-induced alterations in Krebs’ cycle enzyme activities. Rin-5F cells were treated with (0.06–0.3 mM) palmitic acid under normal and high glucose conditions. Activities of ROS-sensitive enzyme, aconitase (**A**), and glutamate dehydrogenase (**B**) were measured as described previously in the Materials and Methods section. Results are expressed as mean +/− SEM of three experiments. Asterisks indicate significant differences (* *p* ≤ 0.05, ** *p* ≤ 0.01, *** *p* ≤ 0.001) relative to untreated control cells under normal glucose condition (NG-C), and triangles indicate significant differences (Δ *p* ≤ 0.05) relative to untreated control cells under high glucose condition (HG-C).

A significant reduction (≈44%) in the activity of aconitase, a ROS-sensitive mitochondrial matrix enzyme, was also observed after treatment with high concentration of palmitic acid (0.3 mM) under normal glucose conditions (Figure 5A). On the other hand, 0.06 mM palmitic acid had no significant effect on aconitase activity. Although high glucose treatment alone caused a moderate decrease in aconitase activity, no further decrease was observed with palmitic acid. This might suggest that high glucose protects aconitase from the deteriorating effects of palmitic acid. Palmitic acid treatment also caused a significant decrease (≈30–60%) in GDH activity in a concentration-dependent manner under normal glucose conditions (Figure 5B). Similarly, under high glucose conditions, high concentration of palmitic acid diminished the activity of GDH (≈20%).

### 3.3. Effects of High Glucose/High Palmitic Acid on Cytosolic Energy Metabolizing Enzymes

**Figure 6:**
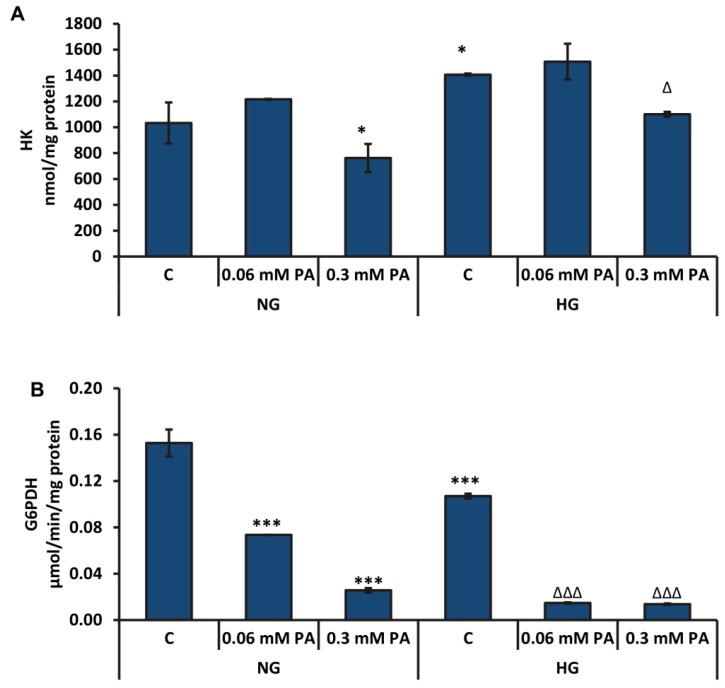
High glucose/high palmitic acid-induced alterations in the activities of hexokinase (HK) and glucose-6-phosphate dehydrogenase enzymes. The activities of hexokinase (**A**) and glucose-6-phosphate dehydrogenase (G6PDH) enzyme (**B**) were measured by a coupled enzyme assay method, as mentioned in the Materials and Methods section. Results are expressed as mean +/− SEM of three experiments. Asterisks indicate significant differences (* *p* ≤ 0.05, *** *p* ≤ 0.001) relative to untreated control cells under normal glucose condition, and triangles indicate significant differences (Δ *p* ≤ 0.05, ΔΔΔ *p* ≤ 0.001) relative to untreated control cells under high glucose condition.

High concentration of palmitic acid (0.3 mM) caused a significant decrease (≈27% and 22%) in the activity of hexokinase (HK), the initial and rate-limiting enzyme of glycolysis, in the presence of normal glucose as well as high glucose, respectively (Figure 6A). High glucose concentration alone appeared to activate the HK enzyme, presumably due to increased glucose uptake and as a mechanism of compensation for the excess glucose in the medium, which was not altered appreciably with low concentration of palmitic acid.

The activity of G6PDH was markedly reduced in a concentration-dependent manner by palmitic acid in the presence of normal glucose and high glucose (Figure 6B). The inhibitory effect was more pronounced under high glucose conditions.

### 3.4. Effects of High Glucose/High Palmitic Acid on Expression of Oxidative Stress and Cell Signalling Markers

**Figure 7:**
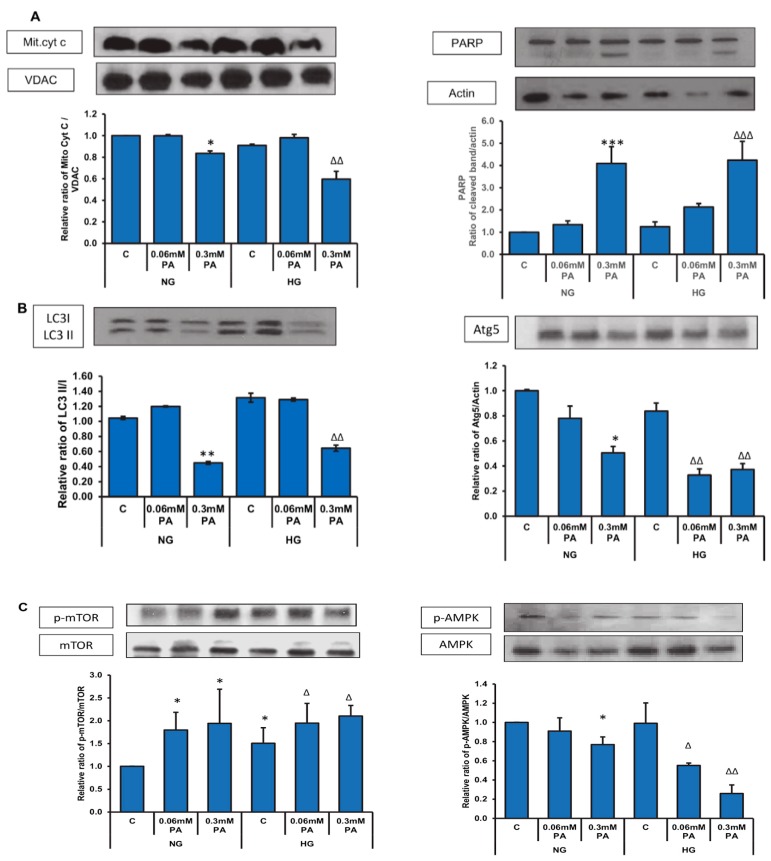
High glucose/high palmitic acid treatment-induced alterations in the expression of apoptotic, autophagy, and signaling proteins. Total extracts (30 µg protein) or mitochondrial extracts (10 µg) from control and treated cells were separated on 12% SDS-PAGE and transferred onto nitrocellulose paper by Western blotting. Mitochondrial cytochrome c (Mit. cyt c) and PARP (**A**), LC3 and Atg5 (**B**), and mTOR and AMPK (**C**) proteins were detected using specific antibodies against these proteins. Beta-actin and VDAC were used as loading controls for post-mitochondrial and mitochondrial extracts, respectively. The quantitation of the protein bands is expressed as relative ratios normalized against the loading control or other specific proteins as appropriate, and histograms are expressed as mean +/− S.E.M of three experiments. The blots shown are representative of three experiments. Asterisks indicate significant difference (* *p* ≤ 0.05, ** *p* ≤ 0.01, *** *p* ≤ 0.001) relative to untreated control cells under normal glucose condition (NG-C), (Δ *p* ≤ 0.05, ΔΔ *p* ≤ 0.01, ΔΔΔ *p* ≤ 0.001) relative to untreated control cells under high glucose condition (HG-C).

Figure 7A shows a decrease in the level of mitochondrial cytochrome c with 0.3 mM palmitic acid with normal as well as high glucose. An increased cleavage of PARP was also observed at this dose with both normal as well as high glucose. 

In consistence, a 50% and 70% decrease in the expression of the autophagy proteins, Atg5 and LC3, respectively, was observed with high concentration of palmitic acid under normal as well as high glucose conditions (Figure 7B). A concomitant increase in the phosphorylation of mTOR, an autophagy inhibitor and decreased phosphorylation of AMPK, the central regulator of cell energy homeostasis and metabolism, was also observed with 0.3 mM palmitic acid, which was more aggravated in the presence of high glucose (Figure 7C). 

### 3.5. Effect of NAC Pre-Treatment on Apoptosis in High Glucose/High Palmitic Acid-Treated Cells 

**Figure 8:**
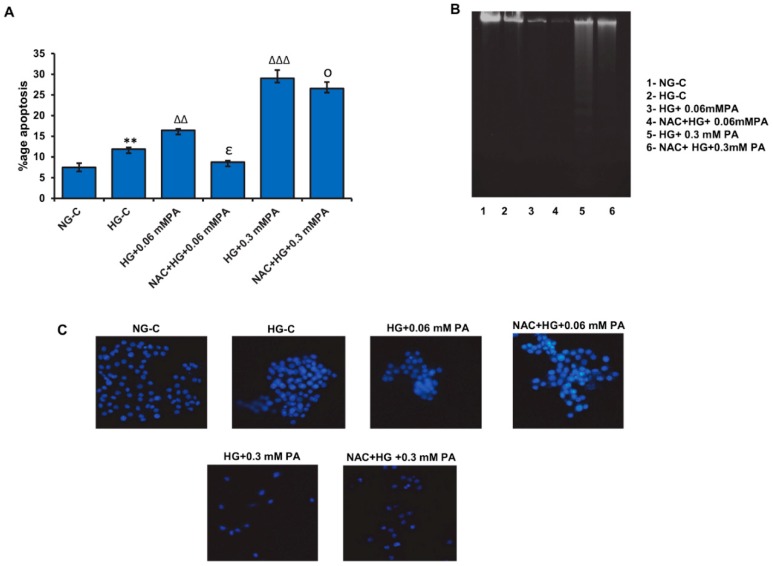
Effect of *N*-acetyl cysteine (NAC) pre-treatment on high glucose/high palmitic acid-induced apoptosis in Rin-5F cells. Apoptosis was measured in Rin-5F cells treated with different doses of palmitic acid under high glucose conditions by flow cytometry. Percentage of apoptotic cells is represented as a histogram (**A**), which is expressed as mean +/− SEM of three experiments. Asterisks indicate significant differences (** *p* ≤ 0.01) relative to untreated control cells under normal glucose conditions, (ΔΔ *p* ≤ 0.01) and (ΔΔΔ *p* ≤ 0.001)relative to untreated control cells under high glucose conditions, (€ *p* ≤ 0.05) relative to 0.06 mM palmitic acid in the presence of high glucose, (○ *p* ≤ 0.05) relative to 0.3 mM palmitic acid in the presence of high glucose. DNA fragmentation was analyzed by agarose gel (2%) electrophoresis and ethidium bromide staining (**B**). Staining of fragmented nuclei of treated cells was performed using Hoechst33342 dye (**C**). Reduced staining with the dye was an indication of apoptotic nuclei Representative slides from three experiments are shown. Original magnification ×200.

As shown before, a significant increase in apoptotic cell death was observed with 0.06 mM palmitic acid in the presence of high glucose, which was further aggravated with 0.3 mM palmitic acid (Figure 8A). NAC pre-treatment caused a moderate reduction in the percentage of cells undergoing apoptosis, suggesting a protective effect of NAC against glucolipotoxicity in these cells.

Increased DNA fragmentation (Figure 8B) and decreased Hoechst-stained nuclei (Figure 8C) were also observed with palmitic acid treatment in the presence of high glucose, which was more pronounced with 0.3 mM palmitic acid. NAC pre-treatment caused a substantial protection against the apoptotic effects of palmitic acid under these conditions.

### 3.6. Effect of NAC Pre-Treatment on the Expression of Apoptotic and Cell Signalling Markers in High Glucose/High Palmitic Acid-Treated Cells

**Figure 9:**
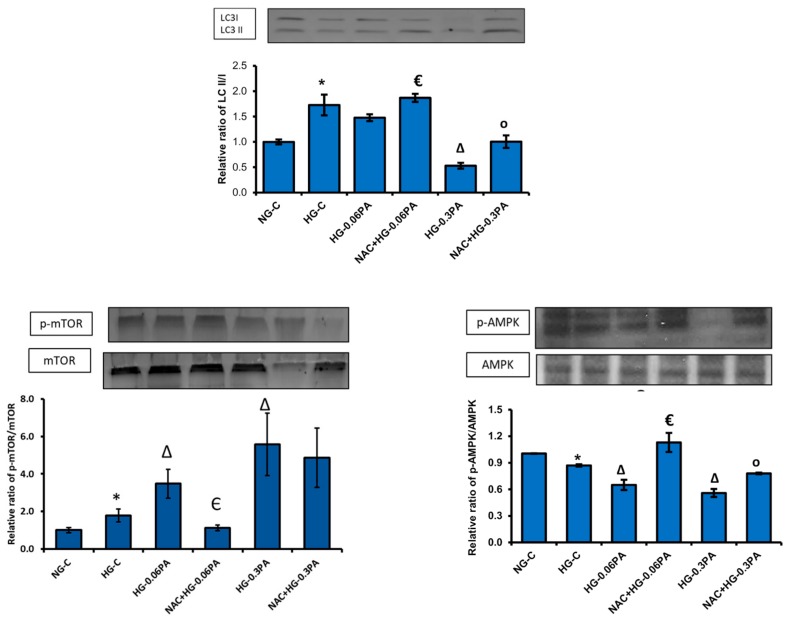
Effect of NAC pre-treatment on the expression of autophagy and cell signaling markers in high glucose/high palmitic acid-treated Rin-5F cells. Total extracts (30 µg protein) from treated cells were separated on 12% SDS-PAGE and transferred onto nitrocellulose paper by Western blotting. LC3, mTOR, and AMPK proteins were detected using specific antibodies against these proteins. Beta-actin was used as loading control. The quantitation of the protein bands is expressed as relative ratios normalized against actin or other specific proteins as appropriate, and histograms are expressed as mean +/− SEM of three experiments. The blots shown are representative of three experiments. Asterisks indicate significant differences (* *p* ≤ 0.05) relative to untreated control cells under normal glucose conditions, (Δ *p* ≤ 0.05) relative to untreated control cells under high glucose conditions, (€ *p* ≤ 0.05) relative to 0.06 mM palmitic acid in the presence of high glucose, (○ *p* ≤ 0.05) relative to 0.3 mM palmitic acid in the presence of high glucose.

NAC caused a significant recovery of the autophagy process by reserving the inhibition of expression of LC3 II induced by high glucose/high palmitic acid, accompanied by a significant-to-moderate inhibition in phosphorylation of mTOR, an autophagy inhibitor (Figure 9), with 0.06 mM and 0.3 mM palmitic acid, respectively. Similarly, NAC pre-treatment recovered the energy homeostasis by blocking the inhibition of AMPK phosphorylation. These results suggest that NAC protects the cells from apoptotic triggers by activating the autophagy self-recovery adaptation processes, as well as by preserving energy homeostasis. 

## 4. Discussion

The deleterious effects of the chronic elevation of glucose and fatty acid levels have been shown to have a significant role in the pathogenesis of numerous diseases, especially metabolic disorders and cancer [1,2,3,4,7]. Several hypotheses have been proposed, including glucotoxicity and lipotoxicity caused by chronic hyperglycemia and dyslipidemia, respectively [2,4,5,8]. A marked synergistic effect of elevated concentrations of glucose and saturated free fatty acids (FFAs) has been shown to induce β-cell death by apoptosis in INS and human islet beta cells [10,31]. Mitochondrial dysfunction has also been shown to be induced by high levels of glucose and free fatty acids [32]. Our previous study on HepG2 cells has also shown that elevated levels of glucose/fatty acid cause severe metabolic and oxidative stress, accompanied by mitochondrial dysfunction and altered energy metabolism [12]. Though numerous studies have been carried out, the exact molecular mechanisms and causative factors are still not clear. This is due to various endogenous and exogenous stimuli affecting the physiological parameters in vivo. Therefore, our aim in the present study was to elucidate the molecular and cellular pathways of pancreatic β-cell toxicity in the presence of high glucose/palmitic acid using an in vitro model of insulin-secreting pancreatic cells, Rin-5F. Furthermore, we aimed to study the cytoprotective effects of NAC, an antioxidant, on the toxicity induced by high glucose/high palmitic acid in Rin-5F cells. Therefore, pancreatic Rin-5F cells were treated with high glucose (25 mM) and high palmitic acid (0.3 mM) for 24 h, alone or in combination, and mitochondrial bioenergetics, energy metabolism in conjunction with cell signaling, and mechanism of cell death were investigated. To study the effects of NAC, cells were treated with 10 mM NAC, 2 h prior to glucose/palmitic acid treatment.

In consistence with numerous other studies, our results indicated increased apoptosis followed by DNA fragmentation, alterations in mitochondrial membrane permeability, and bioenergetics in glucolipotoxicity. These changes were accompanied by alterations in glycolytic and mitochondrial energy metabolism, resulting in inhibition of ATP production. A similar study showed that increasing concentration of palmitic acid conversely correlated with ATP production after palmitate exposure for 24 h in liver cells [33]. Under high glucose conditions, palmitic acid inhibited the activities of the mitochondrial complexes combined with a decrease in ATP production. This could be due to the dampening effect of glucose, which was also observed by Barlow and his colleagues [34]. It has been shown that fatty acids affect the mitochondrial inner membrane by inducing proton permeability [35]. Our study confirmed this finding. We observed increased loss of mitochondrial membrane potential with increasing palmitate concentrations under both normal and high glucose conditions, indicating an increase in membrane depolarization. We also observed a marginal increase in the activities of the mitochondrial respiratory enzymes, complexes I, II/III and IV, by palmitic acid under normal glucose conditions. This increase in complex activities may promote leakage of electrons from the mitochondrial electron transport complexes, contributing to increased ROS production, leading to increased oxidative stress and apoptosis. Consistent with our findings, other researchers have also shown an increase in oxidative stress and mitochondrial membrane depolarization, accompanied by a decrease in ATP content in pancreatic β-cells, Rin-5F, and HIT-T15 under high glucose and high fatty acid conditions [36]. Our recent study on Rin-5F cells also confirmed increased ROS production, followed by increased membrane lipid peroxidation and inhibition of GSH-dependent redox homeostasis, accompanied by the release of pro-inflammatory cytokines [13].

The depolarization of the mitochondrial inner membrane promotes the opening of the permeability transition pore (PTP) [37], which, in turn, causes release of cytochrome c and other pro-apoptotic proteins from the mitochondria, activating the apoptotic cascade [38] leading to cell death. In our study, palmitic acid increased the activities of caspase-9 and -3 in a concentration-dependent manner, accompanied by a decrease in mitochondrial cytochrome c. In addition, an increase in PARP cleavage was observed with high concentration of palmitic acid, which supported the observed DNA fragmentation and apoptosis. 

Our results also demonstrate significant alterations in the Krebs’ cycle enzymes, aconitase, and glutamate dehydrogenase (GDH). Palmitic acid inhibition was more pronounced in the presence of normal glucose. GDH, which catalyzes the reversible deamination of L-glutamate to α-ketoglutarate, is regulated by the cell’s energy state [39]. In our study, we found that palmitic acid caused a decrease in ATP production. This may be a consequence of glutamate influx inhibition, due to a decrease in GDH activity, and ultimately diminished activity of the Krebs’ cycle. This reduction in Krebs’ cycle activity could also be due to an inhibition of glycolytic enzymes, hexokinase, and G-6-P dehydrogenase, after treatment with palmitic acid, as shown in our study. It has been reported that exposure to high concentration of palmitic acid decreased the expression of hexokinase and pyruvate dehydrogenase enzymes and increased the oxidation of palmitoylcarnitine, thus shifting the aerobic metabolism towards the oxidation of fatty acids in the endothelial cells [40]. Other studies have also supported these findings, suggesting an adaptation in energy metabolism, diverting towards anabolism to adapt with the high glucose/fatty acid toxicity [41]. 

Several studies have reported autophagy dysfunction in pathological conditions such as diabetes, cancer, and neurodegenerative diseases [42,43,44]. Apoptosis is down-regulated by autophagy, and apoptosis-associated caspase activation shuts off the autophagy process [45,46]. In our study, we found that high concentrations of palmitic acid decreased the expression of autophagy proteins, Atg5 and LC3, along with increased apoptosis. Consistent with our finding, other investigators found that glucose and palmitic acid exposure inhibit the autophagy turnover in β-cells, leading to apoptotic cell death through activation of mTORC1, an inhibitor of autophagy [47], as confirmed in our study. We hypothesize that the cellular adaptive mechanisms (autophagy) could be overwhelmed in the presence of high concentration of glucose and palmitic acid, pushing the cells into the apoptotic phase. Furthermore, it has been shown that apoptosis accompanied by caspase activation results in cleavage of autophagy proteins, causing inactivation of the autophagy mechanism, resulting in abortion of its cytoprotective action [45].

It has been shown that autophagy is also regulated by mTORC and AMPK [48,49,50]. Studies have shown that nutrient overload activates mTORC1 and inhibits AMPK, and consequently suppresses autophagy [51,52], which is consistent with our study. We also found that NAC pre-treatment resulted in reversal of autophagy suppression by inhibiting mTOR activation and stimulating AMPK. The stimulation of autophagy was confirmed by an increase in the expression of autophagy protein LC3-II. Studies have indicated therapeutic effects of NAC against insulin resistance and type-2 diabetes [53]. NAC has also been shown to exert its antioxidant and anticancer effects by modulating glutathione metabolism and biotransformation of carcinogenic substances in vivo [13,53]. Consistent with the reversal in autophagy suppression by NAC, we also observed decreased apoptosis, as shown by FACS analysis. This was confirmed by reduced DNA laddering and increase in the nuclear Hoechst-stained cells. Our previous study on HepG2 cells [19] also showed that NAC treatment partially protected the oxidative modification of mitochondrial complexes and helped in preserving the mitochondrial function, thus decreasing membrane permeabilization. This could be another reason for the decreased apoptosis observed in the pancreatic cells after NAC treatment.

## 5. Conclusions

**Figure 10:**
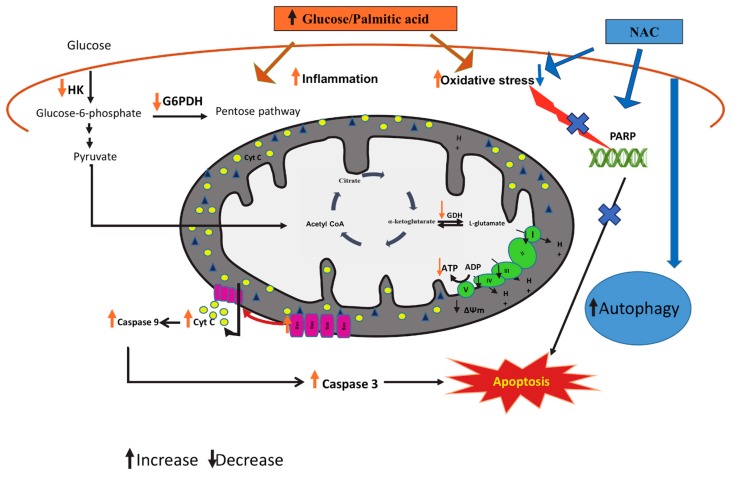
Schematic model representing the cytoprotective mechanisms of NAC in glucolipotoxicity-induced Rin-5F cells. High glucose/high fatty acids have been shown to cause increased oxidative stress, DNA breakdown, and mitochondrial dysfunction, causing activation of caspases and leading to apoptosis. Glucolipotoxicity has also been shown to cause alterations in the expression of autophagy and cell signaling markers. As shown in the model, NAC protects the cells from the glucolipotoxicty-induced mitochondrial and metabolic stress by enhancing autophagy, leading to suppression of apoptosis, thus reducing oxidative stress. GDH: glutamate dehydrogenase, HK: hexokinase, G6PDH; glucose-6-phosphate dehydrogenase, Cyt c; cytochrome c.

Our results suggest that high glucose and high palmitic acid induce cytotoxicity in pancreatic β-cells by stimulating oxidative DNA fragmentation and mitochondrial dysfunction, leading to increased apoptosis (schematic representation shown in Figure 10). The nutrient overload in glucolipotoxicity caused the cells to suppress autophagy and inhibit energy-related catabolic pathways and ATP production. Furthermore, we have also provided evidence that the effects of high glucose and high palmitic acid are attenuated by NAC, an anti-oxidant, suggesting the role of oxidative stress in glucolipotoxicity. This is in consistence with our recent study [13], showing increased oxidative stress and inflammatory responses in Rin-5F cells, accompanied by inhibition of glutathione-dependent redox homeostasis.
